# Supplementary material for: Psychometric properties of the S-Scale: Assessing a psychological mindset that mediates the relationship between socioeconomic status and depression
Source: PLoS One. 2021 Oct 14;16(10):e0258333. doi: 10.1371/journal.pone.0258333 (PMC8516301; doi:10.1371/journal.pone.0258333)
Supplement: S1 Table — English version. (DOC) [file pone.0258333.s001.doc]

**You are now reading a series of statements that people can make about themselves. Some of the statements are positive, others are negative. Please indicate to what extent the following statements apply to you personally.**

|  |  |  | 0 = Does not apply to me at all | 1 | 2 | 3 | 4 = Does apply to me completely |
| --- | --- | --- | --- | --- | --- | --- | --- |
| 1 | LOT-R, Item 3 | If something can go wrong for me, it will. | 0 | 1 | 2 | 3 | 4 |
| 2 | RS-11, Item 5 | I feel that I can handle many things at a time. | 0 | 1 | 2 | 3 | 4 |
| 3 | SWLS, Item 1 | In most ways my life is close to my ideal | 0 | 1 | 2 | 3 | 4 |
| 4 | SHS, Item 1 | In general, I consider myself not a very happy person. | 0 | 1 | 2 | 3 | 4 |
| 5 | LOT-R, Item 9 | I rarely count on good things happening to me. | 0 | 1 | 2 | 3 | 4 |
| 6 | RS-11, Item 3 | Keeping interested in things is important to me. | 0 | 1 | 2 | 3 | 4 |
| 7 | LOT-R, Item 7 | I hardly ever expect things to go my way. | 0 | 1 | 2 | 3 | 4 |
| 8 | SWLS, Item 2 | The conditions of my life are excellent | 0 | 1 | 2 | 3 | 4 |
| 9 | RS-11, Item 9 | I can usually look at a situation in a number of ways. | 0 | 1 | 2 | 3 | 4 |
| 10 | SWLS, Item 4 | So far, I have gotten the important things I want in life. | 0 | 1 | 2 | 3 | 4 |

Note. SWLS = Satisfaction with Life Scale (Diener et al., 1985), SHS = Subjective Happiness Scale (Lyubomirsky & Lepper, 1999), LOT-R = Life Orientation Test Revised (Herzberg & Glaesmer, 2006; Scheier & Carver, 1995), RS-11 = German Resilience Scale (Leppert & Dye, 2002; Schumacher et al., 2005)

**References**

Diener, E. D., Emmons, R. A., Larsen, R. J., & Griffin, S. (1985). The Satisfaction with Life Scale. *Journal of Personality Assessment*, *49*(1), 71–75.

Herzberg, P. Y., & Glaesmer, H. (2006). *Separating Optimism and Pessimism : A Robust Psychometric Analysis of the Revised Life Orientation Test (LOT-R)*. *18*(4), 433–438.

Leppert, K., & Dye, L. (2002). RS–Resilienzskala. In E. Brähler, J. Schumacher, & B. Strauß (Eds.), *Psychodiagnostische Verfahren in der Psychotherapie.* (pp. 295–298). Hogrefe: Göttingen.

Lyubomirsky, S., & Lepper, H. S. (1999). A measure of subjective happiness: preliminary reliability and construct validation. *Social Indicators Research*, *46*, 137–155.

Scheier, M. F., & Carver, C. S. (1995). Distinguishing optimism from neuroticism (and trait anxiety , self-mastery, and self-esteem): A reevaluation of the life orientation test. *Journal of Personality and Social Psychology*, *67*, 1063–1078.

Schumacher, J., Leppert, K., & Gunzelmann, T. (2005). Die Resilienzskala – Ein Fragebogen zur Erfassung der psychischen Widerstandsfähigkeit als Personmerkmal. *Zeitschrift für klinische Psychologie, Psychiatrie und Psychotherapie*, *53*, 16–39.
