# Supplementary material for: Psychometric properties of the S-Scale: Assessing a psychological mindset that mediates the relationship between socioeconomic status and depression
Source: PLoS One. 2021 Oct 14;16(10):e0258333. doi: 10.1371/journal.pone.0258333 (PMC8516301; doi:10.1371/journal.pone.0258333)
Supplement: Attachment — Submitted filename: Review PLOS R2 Psychological Mindset.docx [file pone.0258333.s003.docx]

**Review for Psychometric properties of the S-Scale: Assessing a psychological mindset that mediates the relationship between socioeconomic status and depression.**

**PONE-D-20-39877. R1**

I appreciate the authors for their responses. The revised version of the manuscript addresses many of the issues reviewers’ raised, and the manuscript is now more robust and looks balanced in its strengths and weaknesses.

However, the main concerns were addressed superficially. New measures should improve previous measures in some way or be based on a new construct. The proposed S-scale somehow combines both: it tries to combine previous measures to create a new measure and proposes a new construct (“SES-related *psychological mindset*”).

To represent a significant contribution to the scientific and psychological literature, new measures should be based on different reliability and validity considerations. This manuscript offers evidence of internal consistency (only one type of reliability) and some evidence of validity. However, critical aspects of validity for the proposed uses of the scale, such as incremental validity and content validity, are not discussed.

Content validity issues are very relevant for a new measure trying to measure a new construct but only justified in terms of correlational association with self-reported SES. Furthermore, the content of the retained items is not discussed – how they relate to each other to sum up to the new latent variable.

When addressing incremental validity, the main question is: is the S-Scale necessary? Authors refer that the S-scale is short and can be used in epidemiological surveys. However, the question remains. All the other measures can be shortened to create reduced versions, and some are already short (5 items or less). How much added variance is explained by mixing up items of different constructs and introducing the “psychological mindset/S-Scale” variable. This question is critical and of much interest both for readers and the success of the S-Scale. Does the S-scale explain more variance when exploring if it mediates the association between SES and depression than optimism and life satisfaction? The authors can answer this question using data from Studies 2 and 3.

Cutoff criteria suggested for assessing fit indices still need clarification. More justification for the selected values should be provided. Hu & Bentler criteria, despite cited, recommends other thresholds.

In line 545, the correlation between S-scale and narcissism is minimal (.05), but in table 5 the value is .13***. On the contrary, correlations between S-scale and self-esteem and daily stressors are coincident.

What does it mean that items originally from the same scale were allowed to correlate? Maybe the authors mean they correlated the errors terms? Fit indices of competing models should be provided: with and without correlations between error terms and also modeling original constructs as factors. If the fit of the unidimensionality model is better, more evidence for the utility of the S-scale will be provided.
